# Risk factors for impaired fasting glucose or diabetes among HIV infected patients on ART in the Copperbelt Province of Zambia

**DOI:** 10.1186/s40200-017-0310-x

**Published:** 2017-07-17

**Authors:** Perfect Shankalala, Choolwe Jacobs, Samuel Bosomprah, Michael Vinikoor, Patrick Katayamoyo, Charles Michelo

**Affiliations:** 1 School of Public Health, Department of Epidemiology and Biostatiscs, University of Zambia, P.O Box 5110, Lusaka, Zambia; 2 Family Health International (fhi360), Plot 2374, Farmers Village, ZNFU Complex, Showground’s Area, TiyendePamodzi Road, Off Nangwenya Road, P.O. Box 320303, Lusaka, Zambia; 3 Centre for Infectious Diseases Research in Zambia, 5032 Great North Road, Lusaka, Zambia; 4 Department of Biostatistics, School of Public Health, University of Ghana, Accra, Ghana; 5 Department of Medicine, University of Alabama at Birmingham, Birmingham, USA

**Keywords:** Impaired fasting glucose, HIV, cART patients, Zambia

## Abstract

**Background:**

Africa has a high prevalence of both Human Immunodeficiency Virus and Non Communicable Diseases (NCDs) but in Zambia there are few data on co-morbid NCDs like Diabetes Mellitus (DM) among HIV-infected individuals. We aimed to identify risk factors for impaired fasting glucose or diabetes among HIV-infected Zambians on long-term Combined Antiretroviral Treatment (cART).

**Methods:**

This was a cross sectional study of adult HIV patients in five health facilities of Copperbelt Province in Zambia. HIV/AIDS patients aged 18 years and above, enrolled in care at those health facilities and had been on cART for more than 2 years were included. All patients known to have Diabetes mellitus were excluded from the study. Participants underwent assessment of random blood sugar levels at enrolment and returned the following morning for fasting glucose measured by glucometers. The primary outcome was proportion with impaired fasting glucose or DM. Multivariable logistic regression was used to examine if demographics, time on ART, type of ART regimen, body mass index and baseline CD4 count were predictors of impaired fasting glucose.

**Results:**

Overall (*n* = 270) there were 186 females (69%) and 84 males (31%). The prevalence of impaired fasting blood sugar or diabetes after 8 h of fasting was 15% (95%CI: 11.1, 20.0). Ten percent (26/270) had impaired fasting glucose and 5 % (14/270) had diabetes. Impaired fasting glucose was higher in males than females [AOR = 3.26, (95% CI: 1.15–9.25; *p*-value = 0.03)]; as well as among patients on second line treatment than those on first line [AOR = 3.87 (95% CI 1.16–12.9); *p*-value = 0.03]. In contrast those with less likelihood of impaired fasting glucose included patients with a normal BMI (18.5–24.9) than overweight or obese patients [AOR = 0.09 (95% CI 0.03–0.31; *p*-value < 0.001)]; and participants who had less than 4 diabetes symptoms than those with more than 4 diabetes symptoms [AOR = 0.04 (95% CI 0.02–0.12); *p*-value < 0.001].

**Conclusion:**

We have found high levels of impaired fasting glucose or diabetes among ART patients compared to what is reported in the general population suggesting missed care and support opportunities associated with metabolic imbalance management. There is thus a need to re-package HIV programming to include integration of diabetes screening as part of the overall care and support strategy.

## Background

Diabetes mellitus is a chronic condition affecting approximately 285 million people in the world and the figure is expected to rise by more than 50% in the next twenty years [1–3]. In Zambia, the prevalence of impaired fasting glucose (IFG) or diabetes in the general population is estimated at 4.1% [4, 5] but we do not know the burden in the Human Immunodeficiency Virus/Acquired Immunodeficiency Syndrome (HIV/AIDS) subpopulation.

The consequence of high burden of diabetes among HIV/AIDS patients is cardiovascular diseases and subsequent mortality. Patients on Antiretroviral Treatment (ART) may be more likely to have Non Communicable Diseases (NCD) because the competing risk of opportunistic infection falls and some drugs may induce metabolic disorders. In view of this many ART patients have experienced increase in morbidity and mortality from diabetes mellitus [6, 7]. Although it could reduce mortality, screening for DM has not been broadly implemented in African HIV programs. For example, there are minimal data on blood sugar among HIV patients in Zambia, including the nearly 700,000 who are on prolonged duration of ART. If unchecked, this could potentially complicate clinical management and cART treatment outcomes.

In Senegal the prevalence of diabetes among ART patients is 14.5% which is seemingly higher than what is reported in the general population [8]. Traditional risk factors, such as obesity, ageing and male sex, are important determinants of diabetes [9, 10]. A recent study demonstrated that second line treatment with efavirenz, stavudine and zidovudine increased the risk of incident diabetes [9, 11, 12]. It is thus important to evaluate the possibility of introducing a diabetes screening intervention within the HIV/AIDS care settings especially in resource-constrained countries where most patients are taking these drugs.

To better understand both the epidemiology of Diabetes Mellitus among HIV patients in Zambia and to explore symptom-based screening in the context of routine programmatic care, we asked the question “what is the burden of impaired fasting glucose or diabetes among HIV/AIDS patients on ART in the Copperbelt province of Zambia? We also investigated potential factors associated with impaired fasting glucose or DM among ART patients. We believe that quantifying the burden in this subpopulation would contribute to knowledge in this field to help spur discussion around integration of diabetes screening into HIV programming.

## Methods

### Setting

Copperbelt province is the main mining province of Zambia and has some of the most developed commercial and industrial areas in the country. The province has one of the highest prevalence of HIV/AIDS in Zambia of about 18% and subsequently has a reasonably high number of patients on cART [13]. Family Health International (fhi360) supports the Government Republic of Zambia in delivery of HIV/AIDS programmatic activities in this province and thus has a list of all health facilities where all programmatic activities are undertaken.

### Design & participant selection

This was a cross sectional design conducted among adult ART patients in the Copperbelt province from October to December 2015. Multistage sampling was employed where in the first phase five healthcare facilities with functioning glucometers were conveniently selected from among 250 health facilities. The Five health facilities were Ndola Central Hospital, Thompson General Hospital, Nchanga General Hospital, Twapia Health Centre and Chawama Health Centre. In the second stage we aimed to select participants from among all HIV/AIDS patients aged 18 years and above, enrolled in care at those health facilities and had been on cART for more than 2 years. While we desire to assess the prevalence of DM in the HIV/AIDS patients we deliberately excluded patients with established DM on glucose-lowering drugs. Also, because we defined IFG/DM strictly according to the blood tests these patients may have been missed by this definition and therefore will have little impact on the estimate. The remaining eligible participants were then offered to participant in the study and informed that their participation is voluntary and could opt out without being prejudiced on future care at this facility.

We used the prevalence of impaired fasting glucose in the general population in Zambia estimated at 4.1% in order to estimate a well powered sample size estimation [5]. Further, we expect the burden to be higher in HIV/AIDS subpopulation because of high exposures to predisposing factors [8], for this study we calculated the sample size based on the assumption of 30% prevalence in HIV/AIDS sub-population. We therefore required a minimum total of 326 HIV/AIDS patients with 80% power using Pearson’s chi-squared two-sided 5% level of significance.

For each facility, ART patients were selected at intervals from the sampling frame using systematic sampling procedure. We varied the sampling interval from facility to facility, based on sample size allocations so that facilities with more participants get a proportionally higher number of patients enrolled in the study compared to facilities with fewer patients. We reduced the sampling interval for larger facilities in order to recruit more patients. That is, every 5th for Ndola Central Hospital, 7th for Tompson General Hospital, 6th for Nchanga North General Hospital, 14th for Twapia Health Centre, and 15th for Chawama Health Centre. However, we appreciate that maintaining a constant sampling interval would have been statistically more desirable.

### Data Collection & Laboratory Procedures

Screening checklist was used to collect important clinical and socio-demographic characteristics, including age, sex, marital status, smoking status, body mass index, baseline CD4 count, baseline weight, type of ART regimen and ART duration. In Zambia, first line ART contains fixed dose combination efavirenz, lamivudine or emtricitabine, and tenofovir disoproxil fumarate. Second line contains ritonavir-boosted lopinavir plus 2 nucleoside analogs. The nucleosides are zidovudine + lamivudine for thos on tenofovir in 1st line and are lamivudine or emtricitabine plus tenofovir disoproxil fumarate for those on zidovudine + lamivudine in 1st line. Thereafter All ART patients that met the inclusion criteria and had given informed consent were administered a diabetes symptom screening checklist and consequently underwent a simple random blood sugar test. Patients were then asked to come the following day after 8 h of fasting for glucometer readings to determine impaired fasting blood sugar levels. Validation of glucometers and pre-testing of data collection tool was done before fasting glucose levels were taken. Glucometers were initially used on non-diabetic patients to check for accuracy before actual reading could be taken on study participants. We used Accu check active glucometers and corresponding testing strips. This brand of glucometers comes with precalibration certificates. After collection of 1 drop of whole blood from the fingertip, the device gave blood glucose in mmol/L units. All out of range results suggesting potential diabetes were repeated on gold standard analyser. Nurses and research assistants who were trained on the use of the device performed the finger prick testing and documented the results in the patients’ files and on the research case report form.

### Statistical analysis

The primary outcome was defined as the fraction of patients with either an impaired fasting glucose or diabetes. Impaired fasting glucose was defined as HIV/AIDS patients on ART with blood sugar levels greater than 6.1 mmol/L but less than 7.0 mmol/L, and diabetes defined as HIV/AIDS patients on ART with fasting blood sugar levels greater than 7.0 mmol/L using the World Health Organisation guidelines [14]. The proportion of patients on ART with either impaired fasting sugar or diabetes was disaggregated by socio-demographic characteristics. Fisher’s exact test was used to assess the association of the primary outcome with each characteristic. In a secondary analysis to investigate potential risk factors for impaired fasting glucose or diabetes, we used stepwise backward selection multivariable logistic regression model. The probability of removal was set at 0.2 using likelihood ratio test. Analysis was performed using Stata MP 14 (StataCorp, College Station, Texas, USA).

### Ethical considerations

ERES Converge committee approved the protocol (I.R.B No. 00005948) and the study was on voluntary participation with an option to opt out at any time without giving reasons. Whilst doing this we maintained privacy and confidentiality in line with existing local ethical guidelines. In addition, permission to conduct the study was granted by FHI360 Zambia, Ministry of health (MoH) and the district authorities in charge of ART facilities.

## Results

### Distribution & basic characteristics

A total of 270 ART patients were enrolled, of which 186 (69%) were females and 84 (31%) were males. Overall the median age 46 years (IQR: 38–51) and the median age was 49 years (IQR: 41–55) in males and 45 years (IQR: 37–50) in females. The participant distribution by married status was as follows: married, 60%; single 11% and those who were either widowed or separated were 29%. There were no refusals among the eligible participants. Majority of patients were on first line treatment (88%) and had a median ART duration of 6 years (IQR = 2–12) (Table 1).

**Table 1:** Socio-demographic and Clinical Characteristics of 270 HIV Patients on ART for at least 2 years in Copperbelt province, Zambia

Characteristics	Overall (% of total)	Men (% of total)	Women (% of total)
Age (Years)			
20–34	43 (16)	43 (16)	33 (18)
35–45	95 (35)	95 (35)	71 (38)
45–70	133 (49)	133 (49)	82 (44)
Marital status			
Married	182 (67)	71 (84)	110 (60)
Single	26 (10)	5 (6)	21 (11)
Divorced	23 (9)	3 (4)	20 (11)
Widowed	39 (14)	5 (6)	34 (18)
Body Mass Index (kg/m2)			
< 18.5	31 (11)	8 (10)	23 (12)
18.5–24.9	132 (49)	48 (57)	83 (45)
25–30	43 (16)	13 (15)	30 (16)
> 30	65 (24)	15 (18)	50 (27)
Nadir CD4+ count			
< 200	126 (46)	40 (48)	86 (46)
200–350	75 (28)	23 (27)	51 (27)
350+	71 (26)	21 (25)	49 (26)
Smoking status			
Non smokers	254 (95)	74 (90)	179 (97)
Smokers	13 (5)	8 (10)	5 (3)
Current ART regimen			
1st line ART	238 (88)	70 (83)	168 (90)
2nd line ART	32 (12)	14 (17)	18 (10)
Time on ART (years)			
Median (IQR)	6 (2–12)	6 (4–10)	6 (4–8)
Number of Diabetes symptoms			
0	42 (15)	12 (14)	30 (16)
1–3	185 (68)	57 (68)	127 (68)
4–6	45 (16)	15 (18)	29 (16)
Random Blood Sugar levels (mmol/L)			
< 5.6	142 (52)	38 (45)	103 (55)
5.6–7.7	67 (25)	23 (27)	44 (24)
7.8–11.0	30 (11)	12 (14)	18 (10)
11.1 and above	33 (12)	11 (13)	21 (11)

### Proportions

**Fig. 1:**
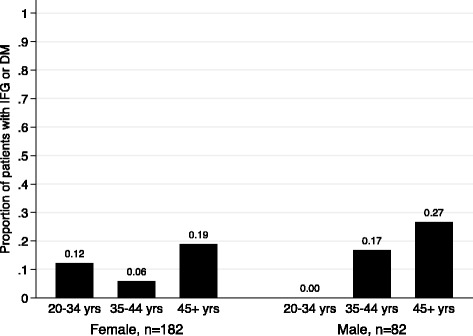
Proportion of patients with impaired fasting glucose (IFG) or diabetics (DM) by age and sex of patients. For female, there were 33, 69, and 80 patients in the age groups 20–34, 35–44, and 45+ respectively. For male, there were 9, 24, and 49 patients in the age groups 20–34, 35–44, and 45+ respectively. There was little evidence of interaction between age and sex on odds of impaired fasting glucose or diabetics (Chi2 = 3.87; *p*-value = 0.144)

The proportion of patients with impaired fasting blood sugar or diabetes was 15% (95%CI: 11.1, 20.0), see Table 2 and of this, 10% (26/270) had impaired fasting glucose and 5% (14/270) had diabetes. The median random blood sugar level in ART patients with a normal fasting blood sugar test was 5 mmol/l IQR (3–7) whereas the median random blood sugar levels for patients with impaired fasting blood sugar was 10 mmol/l (IQR 7–14). Impaired fasting glucose or DM tended to increase with age for both males and female but the increase was more among male (Fig. 1). The highest prevalence was reported among participants who were obese (53%) and those who had 4 or more diabetes symptoms (67%), see Table 2. Participants with a random blood sugar level between 7.8 mmol/L and 11.0 mmol/L recorded the highest combined prevalence of impaired fasting glucose or diabetes.

**Table 2:** Prevalence of impaired fasting glucose or Diabetes by background characteristics

Characteristics	Number (%) with impaired fasting glucose or Diabetes	95%CI	Fisher’s exact *p*-value
Sex			
Male	17 (20.7)	[13.2, 30.9]	0.098
Female	23 (12.6)	[8.5, 18.5]	
*Age (years)*			
20–34	4 (9.3)	[3.4, 22.5]	0.015
35–45	8 (8.6)	[4.3, 16.3]	
45–70	28 (21.7)	[3.6, 29.7]	
Marital Status			
Married	27 (15.2)	[10.6, 21.3]	0.277
Single	3 (11.5)	[3.7, 30.9]	
Divorced	1 (4.6)	[0.6, 27.2]	
Widowed	9 (23.1)	[12.3, 39.0]	
*Body Mass Index*			
< 18.5	1 (3.3)	[0.4, 20.9]	<0.001
18.5–24.9	4 (3.1)	[1., 7.9]	
25–30	2 (4.8)	[1.1, 17.4]	
> 30	33 (53.2)	[40.7, 65.3]	
*Nadir CD4+ count*			
< 200	12 (9.8)	[5.6, 16.4]	0.011
200–350	10 (13.5)	[7.4, 23.5]	
.> 350	18 (26.5)	[17.3, 38.5]	
Current Smoking Status			
Non Smokers	38 (15.3)	[11.3, 20.4]	1.000
Smokers	2 (15.4)	[3.6, 46.7]	
*Current ART Regimen*			
1st line	30 (12.8)	[9.1, 17.7]	0.006
2nd line ART	10 (33.3)	[18.7, 52.2]	
*Number of Diabetes symptoms*			
0	5 (12.8)	[5.4, 27.7]	<0.001
1–3	7 (3.8)	[1.8, 7.8]	
4–6	28 (66.7)	[51.0, 79.3]	
*Random Blood Sugar Levels (mmol/L)*			
< 5.6	**	**	<0.001
5.6–7.7	3 (4.7)	[1.5, 13.7]	
7.8–11.0	25 (92.6)	[74.1, 98.2]	
11.1 and above	12 (37.5)	[22.4, 55.4]	
Total	40 (15)	[11.1, 20.0]

Sex, BMI, ART regimen and number of diabetes symptoms were found to be independently associated with odds of impaired fasting glucose or diabetes (Table 3). Males were about 3 times more likely to have impaired fasting glucose or diabetes compared to females [AOR = 3.26, (95% CI: 1.15–9.25; *p*-value = 0.03)]. See Table 3. Participants with a normal BMI (18.5–24.9) had about 91% reduced odds of impaired fasting glucose or diabetes compared to patients that were overweight or obese [AOR = 0.09 (95% CI 0.03–0.31; *p*-value < 0.0001)]. Patients on second line treatment were about 4 times more likely to have impaired fasting glucose or diabetes compared to patients on first line treatment [AOR = 3.87 (95% CI 1.16–12.92); *p*-value = 0.03]. Participants who had less than 4 diabetes symptoms had about 96% reduced odds of impaired fasting glucose or diabetes compared to patients who had more than 4 diabetes symptoms [AOR = 0.04 (95% CI 0.02–0.12); *p*-value < 0.0001] (Table 3).

**Table 3:** Factors independently associated with odds of impaired fasting glucose or Diabetes among ART patients in urban health facilities in Copperbelt province, Zambia

Characteristics	Adjusted Odds ratio (95CI)	Adjusted Wald *p*-value
*Sex*		
Female	1	0.027^a^
Male	3.26 (1.15, 9.25)	
*Body Mass Index (kg/m2)*		
Overweight/obese	1	<0.001^a^
Normal	0.09 (0.03, 0.31)	
*Current ART regimen*		
1st line	1	0.028^a^
2nd line	3.87 (1.16, 12.92)	
*Diabetes symptoms (number)*		
4+	1	<0.001^a^
< 4	0.04 (0.02, 0.12)	

^a^Variables that were statistically significant at 5% significance level

## Discussion

In this study, we aimed to identify potential risk factor for impaired fasting glucose or diabetes among HIV/AIDS patients on ART in Copperbelt province. We estimated the proportion of impaired fasting glucose or diabetes as 15%. The study also showed that males are at greater odds of impaired fasting glucose or diabetes compared to females whereas those who are overweight or obese were more likely to have impaired fasting glucose or diabetes compared to normal patients. Also, the more diabetes symptoms a patient had the more likely he or she would have impaired fasting glucose or diabetes. We have also found that patients on second line treatment were about 4 times more likely to have impaired fasting glucose or diabetes compared to patients on first line treatment.

We found a significantly higher proportion of ART patients with impaired fasting glucose or diabetes compared to what is reported in the general population. Our finding are similar to the reported prevalence of 14% found in Senegal among ART sub populations [8]. However this maybe contrary to the findings of a study involving patients from seven countries in Latin America who found an overall prevalence of 3.6% for type 2 diabetes [15]. The lower prevalence found in these studies when compared to ours can be explained in part by the relatively younger population and in most cases a shorter duration of ARV exposure. We also suggest that the underlying causes and origin of diabetes in HIV/AIDS patients in our study may differ from that of the general population. We also found that men were more likely to have impaired fasting glucose or diabetes compared to females. Our findings are also consistent with that of a Swiss HIV cohort study, which showed that men were more likely to have impaired fasting glucose compared to women and that the prevalence of diabetes was slightly higher among men than women [16]. The Swiss HIV study also found that the incidence of diabetes in patients receiving ART was 4.42 cases per 1000 person-years of follow-up [16].

BMI was one of the factors independently found to be associated with impaired fasting glucose or diabetes. This was not surprising and is in agreement with literature from elsewhere. A study in Senegal, another sub-Saharan country showed that factors such as higher body mass index (BMI) was associated with higher risks of diabetes [8]. This is consistency with our study may suggest presence of minimal biomarker variability among this population. Nonetheless, it is a plausible and important finding because initiation of ART correlates with rapid weight gain and as such risk reduction strategies for overweight and obese individuals should be of primary focus [10]. We also found that patients who had diabetes symptoms greater than 4 were times more likely to have impaired fasting glucose compared to patients who reported not having any symptoms adjusting for other variables. This is an important finding especially in resource limited settings where laboratory equipment is a challenge, diabetes symptoms screening can be used as a basis for diagnosis and avoid duplication of resources by ensuring that only those at risk are further tested enhance improved care. Similarly, the number of diabetes symptoms as a predictor of impaired fasting glucose or diabetes relates to what the international diabetes federation describes as symptoms of marked hyperglycemia which include polyuria, polydipsia, weight loss, sometimes with polyphagia, and blurred vision [6].

Another observation was the relationship between treatments and impaired fasting blood sugar or diabetes. We found that patients on second line treatment were about 4 times more likely to have impaired fasting glucose or diabetes compared to patients on first line treatment. Our finding relates to other studies that have demonstrated that combination drug regimens including PI which are largely offered as second line treatment are accompanied by impaired glucose tolerance, hyperproinsulinaemia as an indicator for beta-cell dysfunction, and lipid abnormalities as significant risk factors for metabolic conditions [10]. Current treatment with protease inhibitor and nucleoside reverse transcriptase inhibitor containing regimens was associated with the risk of developing type 2 diabetes [16]. In our study however, we didn’t analyse the specific combinations of ART regimens that the study participants were exposed to.

It is possible that the estimates that we have found in this study may not be accurate and biased by sampling errors. Firstly we realise that this is a highly selected group in which metabolic disorders have been described elsewhere [17–20]. Secondly, this selection is further highlighted in that this is a hospital-based study that was focussed on clients who come to the facility for HIV care and support. Thirdly, the selected study site is an urban site where anecdotal information suggests presence of higher burden of metabolic disorders even among groups without HIV infection. Fourthly, the facility site selection was guided by presence of glucometer and not demographic or other clinical information that may be of relevance in metabolic assessments. Fifthly, we recognise some methodological limitations associated with the use of glucometers for measuring fasting blood sugar, which could have underestimated our results because glucometers are sensitive to calibrations. It is, therefore, possible that high blood sugar levels could not be read. This could have potentially infused measurement bias in our study. Also, we would have desired to perform sub-group analysis by sex but our study had low statistical power. Oral glucose tolerance test (OGTT) was not performed to further diagnosed diabetes. This could have potentially contributed to under diagnosis of diabetes in this population. The other limitation is that it was hard to ascertain whether patients had undergone 8 h of fasting despite measures being put in place prior to fasting blood sugar test.

When all this is considered, it is possible that the estimates found in our study participants may be different. The magnitude and direction of this bias may be difficult to estimate using the data that we collected. However, we argue that metabolic disorders have been reported in HIV infected clients on ART elsewhere and in Zambia [8, 11, 12, 17, 18]. This body of literature suggests presence of associated glucose impairment and diabetes which has been assumed to be higher than the general population. In this regard and notwithstanding the presence of selection and measurement biases that may be inherent in our small sample, we argue that this may even be an under estimate of the actual burden. We are thus persuaded to believe our observed high levels of impaired fasting glucose and diabetes among ART patients compared to what is reported in the general population. This clearly suggests missed care and support opportunities especially in settings with limited laboratory measurement capabilities which further signals potentials for poor management of clients with metabolic imbalance and associated clinical challenges.

## Conclusions

We have found high levels of impaired fasting glucose or diabetes among ART patients compared to what is reported in the general population suggesting missed care and support opportunities associated with metabolic imbalance management. There is thus a need to re-package HIV programming to include integration of diabetes screening as part of the overall care and support strategy. Notwithstanding the limitations, we believe that this study has helped in making an initial step to quantify the burden of Non communicable diseases such as diabetes among ART patients and this will help in advocating for more sharpened responses which advocates for the integration of NCDs within ART platforms. This could be critically important in reducing the associated burden. Nonetheless there is still need to invest in additional research with larger sample size so as to increase the evidence critical in primary care strategies which directly impact on secondary care and support programmes.

## Abbreviations

AIDS: Acquired Immunodeficiency Syndrome
ART: Antiretroviral Treatment
BMI: Body Mass Index
CART: Combined Antiretroviral Treatment
CD4: Cluster Differentiation
DM: Diabetes Mellitus
HAART: Highly Active Antiretroviral therapy
HIV: Human Immunodeficiency Virus
IDF: International Diabetes Federation
IFG: Impaired Fasting Glucose
NCDs: Non-Communicable Diseases
PIs: Protease Inhibitors
WHO: World Health Organization
ZDHS: Zambia Demographic and Health Survey
ZPCT II: Zambia Prevention and care treatment project
